# AMP-Activated Protein Kinase as a Reprogramming Strategy for Hypertension and Kidney Disease of Developmental Origin

**DOI:** 10.3390/ijms19061744

**Published:** 2018-06-12

**Authors:** You-Lin Tain, Chien-Ning Hsu

**Affiliations:** 1 Departments of Pediatrics, Kaohsiung Chang Gung Memorial Hospital and Chang Gung University College of Medicine, Kaohsiung 833, Taiwan; tainyl@hotmail.com; 2 Institute for Translational Research in Biomedicine, Kaohsiung Chang Gung Memorial Hospital and Chang Gung University College of Medicine, Kaohsiung 833, Taiwan; 3 Department of Pharmacy, Kaohsiung Chang Gung Memorial Hospital, Kaohsiung 833, Taiwan

**Keywords:** AMP-activated protein kinase, developmental origins of health and disease (DOHaD), hypertension, kidney disease, nutrient-sensing signals, oxidative stress, renin-angiotensin system

## Abstract

Suboptimal early-life conditions affect the developing kidney, resulting in long-term programming effects, namely renal programming. Adverse renal programming increases the risk for developing hypertension and kidney disease in adulthood. Conversely, reprogramming is a strategy aimed at reversing the programming processes in early life. AMP-activated protein kinase (AMPK) plays a key role in normal renal physiology and the pathogenesis of hypertension and kidney disease. This review discusses the regulation of AMPK in the kidney and provides hypothetical mechanisms linking AMPK to renal programming. This will be followed by studies targeting AMPK activators like metformin, resveratrol, thiazolidinediones, and polyphenols as reprogramming strategies to prevent hypertension and kidney disease. Further studies that broaden our understanding of AMPK isoform- and tissue-specific effects on renal programming are needed to ultimately develop reprogramming strategies. Despite the fact that animal models have provided interesting results with regard to reprogramming strategies targeting AMPK signaling to protect against hypertension and kidney disease with developmental origins, these results await further clinical translation.

## 1. Introduction

Hypertension and kidney disease have a significant impact on morbidity and mortality worldwide. Hypertension and kidney disease can be the cause and consequence of one another. Importantly, both disorders can originate in early life. Kidneys play a key role in blood pressure (BP) regulation. The developing kidney is highly vulnerable to environmental effects in fetal and infantile life, leading to long-term programming effects on the morphology and functioning of the kidney [1,2]. Adverse renal programming increases the risk for developing hypertension and kidney disease in adulthood [3]. This notion has become a globally recognized concept as developmental origins of health and disease (DOHaD) [4]. Conversely, the DOHaD concept also allows reprogramming [5], a strategy aimed at reversing the initial programming processes prior to the onset of hypertension and kidney disease, in order to shift therapeutic interventions from adulthood to early life. A growing body of evidence suggests that AMP-activated protein kinase (AMPK) plays a decisive role in the normal renal physiology and pathogenesis of hypertension and kidney disease [6,7]. Based on the two aspects of the DOHaD concept, this review will first present the evidence for the link between AMPK signaling and programming mechanisms that may lead to hypertension and kidney disease of developmental origin, with a focus on the kidney. This will be followed by potential pharmacological interventions targeting AMPK signaling that may serve as reprogramming strategies to halt the growing epidemic of hypertension and kidney disease.

## 2. AMP-Activated Protein Kinase in the Renal System

### 2.1. The Structure and Function of AMP-Activated Protein Kinase

AMPK is a phylogenetically conserved, ubiquitously expressed serine/threonine protein kinase. AMPK is a heterotrimer, composed of an α (α1, α2) catalytic subunit, a regulatory and structurally crucial β (β1, β2) subunit, and a regulatory γ (γ1, γ2, γ3) subunit [8,9,10]. These isoforms are encoded by distinct genes and differentially expressed, and have unique tissue-specific expression profiles, creating the potential to generate a diverse collection of 12 αβγ heterotrimer combinations. AMPK has a diverse range of biological functions, including cellular energy homeostasis, glucose metabolism, lipid metabolism, protein synthesis, redox regulation, mitochondria biogenesis, autophagy, ion transport, tumor suppression, anti-inflammation, and nitric oxide (NO) synthesis [8,9,10,11]. The structure and function of these different isoforms have been reviewed in detail previously [8,9,10], and it is not within the scope of the current review to thoroughly outline these further. However, for the purposes of the discussion below, it is important to note that activation of AMPK leads to the biological functions that are linked to renal pathophysiology.

### 2.2. Regulation of AMP-Activated Protein Kinase AMP-Activated Protein Kinase in the Kidney

AMPK is strongly expressed in the kidney, where it is involved in diverse physiological and pathologic processes, including sensing cellular energy status, sodium and ion transport, podocyte function, BP control, the epithelial-to-mesenchymal transition, and NO production [7,8,9,10,11]. In the rat kidney, α1 and β1 subunits are predominant [7,11]. Except for the muscle-specific γ3 isoform, both γ1 and γ2 subunits are similarly expressed in the kidney. However, little is known regarding the differences in AMPK subunit expression between different cell types within the kidney.

The activity of AMPK is mainly regulated by the AMP and adenosine triphosphate (ATP) ratio. AMPK is activated both allosterically and by post-translational modifications. The most well-defined mechanisms of AMPK activation are phosphorylation at αThr^172^ by upstream AMPK kinases and by AMP or adenosine diphosphate (ADP) binding to the γ subunit. So far, at least three kinases and three phosphatases have been identified as upstream AMPK-activating kinases, including liver kinase B1 (LKB1), TGFβ-activated kinase 1 (TAK1), Ca^2+^-/calmodulin-dependent protein kinase β (CaMKKβ), protein phosphatase 2A (PP2A), protein phosphatase 2C (PP2C), and Mg^2+^-/Mn^2+^-dependent protein phosphatase 1E (PPM1E) [12]. Additionally, AMPK can also be regulated by intracellular calcium and oxidant signaling, as well as extracellular signaling like hormones and cytokines [13]. Furthermore, AMPK is the target of a growing number of pharmacological activators [14].

**Figure 1:**
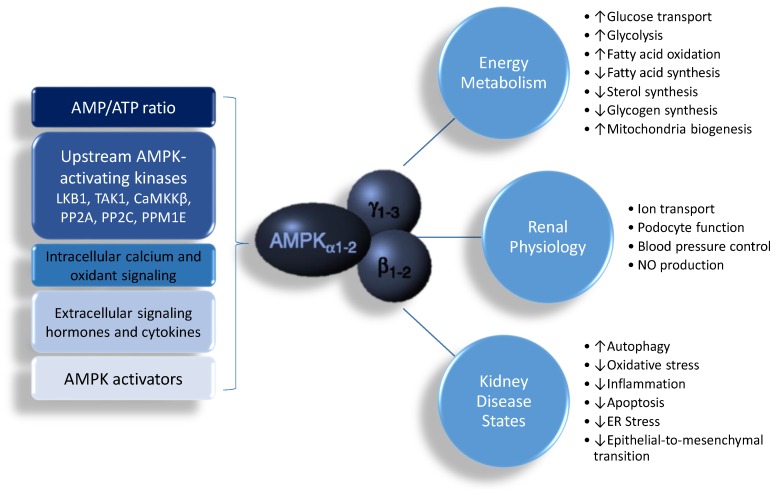
Schematic representation of AMP-activated protein kinase (AMPK) actions and its biochemical functions in the kidney. ↑ = increased. ↓ = decreased.

AMPK has transcriptional effects on numerous enzymes that mediate cellular energy metabolism. AMPK can induce mitochondrial biogenesis by activating the peroxisome proliferator-activated receptor-γ (PPARγ) coactivator-1α (PGC-1α), either directly or through the silent information regulator transcript 1 (SIRT1) [14,15]. Additionally, AMPK and SIRT1 can mediate phosphorylation and deacetylation of PGC-1α, respectively [15], to regulate the expression of PPAR target genes. As reviewed elsewhere, several PPAR target genes contribute to renal programming and hypertension of developmental origins, such as *Sod2*, *Nrf2*, *Sirt7*, *Ren*, *Nos2*, *Nos3*, and *Sgk1* [16]. Another important downstream effect of AMPK is the inhibition of the mammalian target of rapamycin (mTOR). Both AMPK and mTOR can oppositely regulate unc-51-like kinase 1/2 (ULK1/2) activity by phosphorylation to mediate autophagy, a cellular catabolic process in which key organelles are transported to lysosomes for degradation. In addition to activating ULK1/2, AMPK can promote autophagy through SIRT1 by de-acetylating several autophagy-related proteins [17]. Given that autophagy is involved in the pathogenesis of many kidney diseases, and that AMPK regulates autophagic protection against kidney injury, AMPK is becoming a potential target for kidney disease therapy [18]. Additionally, AMPK has been shown to exert anti-inflammatory, antioxidant, and anti-apoptosis effects. Moreover, AMPK regulates many sodium and ion transport proteins in the renal tubular cells, including the epithelial Na^+^ channel (ENaC) [19], the Na^+^–K^+^–2Cl^−^ cotransporter (NKCC2) [20], Na^+^/K^+^-ATPase (NaKATPase) [21], the vacuolar H^+^-ATPase (V-ATPase) [22], and others [7,11,23]. The activation of AMPK and its biochemical pathways are illustrated in Figure 1.

### 2.3. AMP-Activated Protein Kinase in Hypertension and Kidney Disease

Emerging evidence suggests that dysfunction in the AMPK signaling pathway is involved with the development of various cardiovascular diseases, including hypertension [6]. Despite several AMPK activators having been assessed in a number of human studies, interventions necessary to provide a reprogramming strategy and prove causation remain undeveloped. It is for this reason that much of our knowledge of potential mechanisms of renal programming, the impacts of AMPK in renal programming, and reprogramming strategies targeting AMPK signaling come from studies using animal models.

In a genetic hypertension model of a spontaneously hypertensive rat (SHR), AMPK activation was reduced in the aorta of the SHR, while 5-aminoimidazole-4carboxamide riboside (AICAR), a direct AMPK activator, lowered BP [24]. Our previous report showed that metformin, a known AMPK activator, blocks the development of hypertension in SHRs, which is associated with increased renal NO production [25]. Like metformin, activation of AMPK by perinatal resveratrol supplementation has been shown to mitigate the development of hypertension in adult SHRs [26]. Additionally, we recently found that maternal plus post-weaning high-fat diets induced hypertension and reduced renal cortical protein levels of phosphorylated AMPK2α in offspring kidneys, which was prevented by resveratrol therapy [27]. Similarly, AMPK activator metformin was reported to protect adult offspring against the developmental programming of hypertension induced by a maternal plus post-weaning high-fat diet [28]. However, the reprogramming effects of the AMPK activator have not been fully assessed in other developmental programming models for hypertension. Therefore, further investigation is needed to reveal the precise role of AMPK in hypertension of developmental origins, especially the reprogramming effects of AMPK activation.

Apart from its role in the development of hypertension, as mentioned above, there have been many studies on the effects of AMPK in kidney diseases, notably diabetic nephropathy, autosomal dominant polycystic kidney disease, subtotal nephrectomy, lupus nephritis, and renal fibrosis [7]. Although AMPK signals have been studied in established kidney diseases, so far there remains a lack of data on the role of AMPK in renal programming and kidney disease of developmental origin. 

## 3. Common Mechanisms Link AMP-Activated Protein Kinase to Renal Programming

Despite the fact that several organ systems can be programmed in response to adverse environmental exposures in early life, renal programming is considered to be decisive in the development of hypertension, as well as kidney disease [3,5,29,30]. Thus far, a number of proposed mechanisms, including dysregulation of the renin–angiotensin system (RAS), impaired sodium transporters, a nutrient-sensing signal, and oxidative stress have been linked to renal programming [3,5,29,30]. Each mechanism related to AMPK signaling will be discussed in turn. 

### 3.1. Renin–Angiotensin System

The RAS is a central regulator of BP and renal function. The RAS consists of two opposing axes: the angiotensin converting enzyme (ACE)-angiotensin (Ang) II type 1 receptor (AT1R) classical axis, mediated primarily by Ang II; and the ACE2-angiotensin-(1–7)-Mas receptor axis, mediated mainly by Angiotensin-(1–7) [31]. In contrast to ACE, ACE2 appears to adjust the angiotensin II type 2 receptor (AT2R) and the angiotensin (1–7) receptor Mas in a way that opposes the development of hypertension [31]. Over-activation of the classical RAS leads to hypertension and kidney disease [31]. Notably, this hormone-signaling pathway controls kidney development [32]. Both RAS axes and the above-mentioned RAS components have been linked to fetal programming [33,34,35,36]. There is a biphasic response with reduced classical RAS expression at birth that increases with age. Early-life renal programming might activate the classical RAS, leading to hypertension and kidney disease development in later life. Conversely, early blockade of the classical RAS has been shown to prevent the development of hypertension and kidney disease [37,38,39]. Decreased renal AMPK expression has been found in uninephretomized rats with the activation of the RAS [40], which was prevented by the blockage of the RAS. AMPKα2 knockout mice expressed high ACE levels, resulting in vasoconstriction [41]. Conversely, prenatal metformin therapy has been shown to restore the maternal high-fructose plus post-weaning high-fat diet-induced increases of RAS components *Ren*, *Atp6ap2*, *Agt*, *Ace*, and *Agtr1a* in the kidney cortex, resulting in protection from hypertension [28]. Given that resveratrol, an indirect AMPK activator, was reported to exert its protective effects in association with increased expression of the AT2R and Mas receptors [42], further studies are required to determine whether AMPK has a role in activation of the ACE2-angiotensin-(1–7)-Mas receptor axis and AT2R to prevent hypertension, and whether this contributes to the reprogramming effects of AMPK activators. Nevertheless, the detailed mechanisms underlying the modulation of RAS by AMPK and its contributions to protection from programmed hypertension kidney disease still await for further study in different models of developmental programming. 

### 3.2. Sodium Transporters

Hypertension and kidney disease of developmental origin have been associated with enhanced sodium reabsorption, attributed to the increased expression of sodium transporters [1,5,29,30]. Several adverse environmental impacts during early life leading to predisposition toward impaired sodium transporters have been reported, including maternal low-protein diet, maternal high-fat diet, maternal exposure to continuous light, and prenatal glucocorticoid exposure [1,5,29]. Several sodium transporters have been identified in the programming processes, including Na^+^/Cl^−^ cotransporter (NCC), type 3 sodium hydrogen exchanger (NHE3), NKCC2, and NaKATPase. We previously showed that a maternal high-fructose diet plus a postnatal high-salt diet increased renal cortical protein levels of NKCC2, NHE3, and NCC in a two-hit model of programmed hypertension [43]. Notably, AMPK regulates several sodium transporters, such as NKCC2 and NaKATPase, which may account for its beneficial effects on hypertension and kidney disease. However, there are as yet no studies examining the role of AMPK in sodium transporters in the kidneys from animal models of programmed hypertension and kidney disease. 

### 3.3. Nutrient-Sensing Signals

Nutrient-sensing signals regulate fetal metabolism and development in response to maternal nutritional input. AMPK is a well-known nutrient-sensing signal [44]. In addition to AMPK, known nutrient-sensing signals exist in the kidney, including SIRT, PPARs, PGC-1α, and mTOR [44]. The interplay between AMPK and other nutrient-sensing signals, driven by early-life input, can regulate PPARs and their target genes, thereby promoting programmed hypertension and kidney disease [16,45]. Our previous work demonstrated that resveratrol, an AMPK activator, prevents the development of hypertension programmed by maternal plus post-weaning high-fructose diets, via regulation of nutrient-sensing signals [46], supporting the notion that nutrient-sensing signals might be a common mechanism underlying the pathogenesis of hypertension and kidney disease of developmental origin. Since AMPK is a crucial hub for the nutrient-sensing signals network, further studies are required to determine whether AMPK has a role in the regulation of renal programming, and whether AMPK activators can serve as reprogramming strategies to prevent the developmental programming of hypertension and kidney disease. 

### 3.4. Oxidative Stress

Oxidative stress is an oxidative shift characterized by an imbalance between oxidants (e.g., reactive oxygen species (ROS)) and antioxidants, in favor of oxidants. The developing fetus is highly vulnerable to oxidative stress damage, due to its low antioxidant capacity [47]. As reviewed elsewhere [29,45], numerous pre- and peri-natal inputs have been linked to renal programming attributed to oxidative stress, including imbalanced maternal nutrition, maternal diabetes, preeclampsia, prenatal hypoxia, maternal nicotine exposure, maternal inflammation, prenatal glucocorticoid exposure, and a high-fat maternal diet. Conversely, some reprogramming interventions have targeted antioxidants in order to reduce oxidative stress, and accordingly, prevent hypertension and kidney disease of developmental origin [30]. As nutrient-sensing is interconnected with redox regulation, AMPK has a key role in regulating antioxidant defense during oxidative stress. AMPK has been reported to upregulate several antioxidant genes, such as superoxide dismutase (SOD), uncoupling protein 2 (UCP2), and nuclear factor erythroid-2-related factor (NRF2) [48]. Additionally, AMPK activation was shown to suppress nicotinamide adenine dinucleotide phosphate (NADPH) oxidase, a primary source of ROS [49]. Furthermore, AMPK promotes autophagy. Since mitochondria are another major source of ROS within cells, activation of mitochondrial autophagy driven by AMPK is also beneficial for reducing oxidative stress. Thus, these findings suggest that the interplay between AMPK and oxidative stress contributes to programmed hypertension and kidney disease. 

All of these observations demonstrate a close link between AMPK and other hypothetical mechanisms involved in renal programming. Nevertheless, there remains no definite conclusion that AMPK plays a central role on mediating other mechanisms leading to hypertension and developmental kidney disease.

## 4. Reprogramming Strategy Targeting AMP-Activated Protein Kinase Signaling

Reprogramming strategies to counterbalance the programming processes that have been employed range from nutritional intervention and lifestyle modification to pharmacological therapy [1,5,50]. Currently, a variety of therapeutic regimens have been reported to either activate or inhibit AMPK activity and its downstream signaling pathway. Since AMPK inhibition, either by AMPK silencing or AMPK inhibitors (e.g., compound C), contributes to hypertension [51,52], treatment modalities for AMPK activation have become more attractive reprogramming strategies. Both indirect and direct AMPK activators have been studied in established hypertension and kidney disease [6,13,18,53]. Modulators that cause AMP or calcium accumulation without a direct interaction with AMPK are classified as “indirect AMPK activators”. Several kinds of indirect AMPK activators have been studied in relation to cardiovascular and kidney disease [13], including metformin, resveratrol, thiazolidinediones (TZDs), polyphenols, berberine, ginsenoside, α-lipoic acid, quercetin, and so on. On the other hand, direct AMPK activators induce conformational changes in the AMPK complex through direct interaction with a specific subunit of AMPK. While some are potent pan-activators (e.g., AICAR) for all 12 heterotrimetric AMPK complexes, others show isoform-specific activations for the α1 (e.g., compound-13), β1 (e.g., PF-06409577 and PF-249), β1/β2 (e.g., GSK621), or γ1 isoforms (e.g., PT-1) [13,48]. However, present knowledge of AMPK activators in the kidney is significantly less advanced than that for other organs, such as the liver, muscles, and heart. So far, only one report has shown that selective AMPKβ1 activators PF-06409577 and PF-249 protect against kidney damage in a rat model of diabetic nephropathy [54]. In the current review, we will primarily be limited to pharmacological therapies aimed at AMPK signaling as a reprogramming strategy to prevent hypertension and kidney disease of developmental origin. Of note, pharmacotherapies will be narrowly restricted to those beginning prior to the onset of hypertension and kidney disease. SHRs, for example, reveal a rise in BP starting from six weeks of age, and a steep increase between 6 and 24 weeks. We therefore restrict our discussion mainly to therapies starting before six weeks of age, using the SHR model. These AMPK activation modalities are listed in Table 1 [25,26,27,28,46,55,56,57,58,59,60,61,62]. Because the field of DOHaD research is beginning to emerge, this list is by no means complete and is expected to grow rapidly.

### 4.1. Metformin

Metformin, the most commonly prescribed first-line antidiabetic drug in the world, exerts its beneficial effects primarily by AMPK activation. Despite a growing body of evidence indicating the protective effects of metformin in established cardiovascular and kidney diseases [63,64], only a few studies have been conducted to explore its reprogramming effects on programmed hypertension and kidney disease. Early metformin treatment in the pre-hypertensive stage blocks the development of hypertension in SHRs [25]. Additionally, maternal metformin therapy protects adult offspring against the developmental programming of hypertension induced by a maternal plus post-weaning high-fat diet [28]. However, a concern raised by these studies is that AMPK-independent effects on metformin were also reported. A better understanding of the AMPK-dependent and -independent mechanisms responsible for the protective effects of metformin on programmed hypertension and kidney disease is therefore warranted.

### 4.2. Resveratrol and Other Polyphenols

Polyphenols are a large group of phytochemicals found in plant-based food. Resveratrol is a naturally occurring polyphenol phytoalexin. It has been considered to have cardiovascular protective effects, including against hypertension [65]. Mechanisms of activation of AMPK by resveratrol appear to elevate AMP levels and inhibit mitochondrial ATP production. Early resveratrol therapy mitigates the development of hypertension in adult SHRs of both sexes [26,55,56]. Using the prenatal hypoxia and postnatal high-fat diet rat model, post-weaning resveratrol treatment protects adult offspring against programmed hypertension [57]. Our previous report showed that a maternal plus post-weaning high-fat diet induced hypertension and reduced protein levels of phosphorylated AMPK2α in the offspring kidney cortex, which resveratrol therapy prevented [27]. Additionally, early post-weaning resveratrol therapy was reported to prevent the development of hypertension of adult offspring exposed to maternal and post-weaning high-fructose consumption [46]. 

Interestingly, increased AMP levels and ATP depletion leading to uric acid production have been demonstrated as key mediators in the pathogenesis of fructose-induced metabolic syndrome and hypertension [66]. Our previous study showed that the mechanisms underlying the development of hypertension in offspring exposed to maternal high-fructose consumption are different from those in adult rats fed with a high-fructose diet [67]. This may explain why resveratrol increases AMP levels to activate AMPK, resulting in a beneficial effect on the offspring’s BP. However, the reprogramming effect of maternal resveratrol on kidney disease has not been fully assessed in developmental programming models. Of note is that resveratrol has multifaceted biological functions; however, to what extent does its reprogramming effect on hypertension and kidney disease can be attributed to AMPK activation deserves further elucidation.

In addition to resveratrol, several polyphenols are capable of activating AMPK, including quercetin, genistein, epigallocatechin gallate, anthocyanin, magnolol, berberine, and so on. Early treatment with magnolol, berberine, or genistein offered protective effects against programmed hypertension in adult SHRs [58,59,60], whereas quercetin did not [68]. While many polyphenols are antioxidants and exert beneficial effects on oxidative stress-related disorders [69], evidence for their reprogramming effects on hypertension and kidney disease as AMPK activators is equivocal. A better understanding of the differential mechanisms of various polyphenols in the prevention and treatment of programmed hypertension and kidney disease is therefore warranted.

### 4.3. Thiazolidinediones

Thiazolidinediones (TZDs) are a class of insulin-sensitizing drugs, including pioglitazone, rosiglitazone, and troglitazone. TZDs exert their effects mainly by activating PPARγ. They are also known to act in part through AMPK activation. TZDs that activate AMPK are associated with the accumulation of AMP. Treatment with pioglitazone and rosiglitazone prior to the onset of hypertension can be protective in SHRs, by attenuating the development of hypertension [61,62]. However, another study showed that perinatal pioglitazone treatment fails to confer antihypertensive or renoprotective effects in adult fawn-hooded hypertensive rats [70]. Thus, further examination is required to understand the protective effects of TZDs in programmed hypertension and kidney disease, which are exerted mainly via AMPK or PPARγ signaling pathway.

### 4.4. Others

Despite progress made in recent years in discovering direct AMPK activators [13,53], little is known regarding their reprogramming effects on hypertension and kidney disease of developmental origins. The first direct AMPK activator, AICAR, has been reported to lower BP in adult SHRs [24]. However, the reprogramming effect of AICAR on programmed hypertension and kidney disease has not been examined yet in developmental programming models. Additionally, oxidative modification of the AMPKα subunit appears to be a major mechanism by which AMPK is activated under conditions of oxidative stress. Therefore, any modulators that induce intracellular ROS generation might serve as AMPK activators. Furthermore, it is of great importance to understand the interplay between the AMPK signaling and other mechanisms underlying renal programming; the application of reprogramming strategies targeting the above-mentioned mechanisms is also feasible for early intervention.

## 5. Conclusions and Future Perspectives

Hypertension and kidney disease in adult life can be programmed by early-life input. This concept opens a new window for preventing or delaying the onset of hypertension and kidney disease via reprogramming. Studies in short-lived animals, with controlled interventions across their lifespan, have provided interesting results from reprogramming interventions to prevent programmed hypertension and kidney disease via targeting AMPK signaling.

Regardless of recent advances in pharmacotherapies for hypertension and kidney disease, only a few studies have targeted their potential for reprogramming. In the current review, the beneficial effects of these treatments are all coming from indirect AMPK activators that are known to act in both an AMPK-dependent and -independent manner. There remains a lack of data regarding AMPK isoforms, specific knockdown models, or direct AMPK activators for renal programming and developmental programming of hypertension. Using modern genomic techniques [71,72], the identification of nephron segment-specific pathways regulated by AMPK isoforms will be an emerging area of interest. Additionally, another question raised from the current review is that the follow-up periods after the cessation of treatment in most cited reprogramming studies was relatively short. Of note is that the reprogramming effects of some perinatal interventions seem to persist beyond six months of age in female, but not male, SHRs [73,74]. Since sex differences appear in AMPK signaling [75], we must determine the long-term effects of AMPK activators in different programming models, and whether there is a sex-dependent response. Furthermore, a major concern in the translation from animal model to human use of AMPK activators is the activators’ still-unknown adverse effects. Because pharmacological activation of AMPK is required to reach a specific target tissue, off-target effects may counter the therapeutic effects we are aiming for. For example, one possible effect of AMPK being off-target is the stimulation of the hypothalamus to increase food intake, despite the fact that our target organ is the kidney. 

The evidence of the reprogramming effects of AMPK is just the beginning of the field. It is worth noting that instead of fully elucidating the potential mechanisms, these studies pointed out several key mechanisms linking AMPK to renal programming. It is clear that better understanding of the isoform- and tissue-specific effects of AMPK for programmed hypertension and kidney disease are required before a reprogramming strategy targeting AMPK could be translated from animal studies to human trials.

## Figures and Tables

**Table 1:** Reprogramming strategy targeted on AMPK signaling in animal models of programmed hypertension and kidney disease.

Animal Models	Gender/Species	Age at Evaluation	Dose and Period of Treatment	Reprogramming Effects	Ref.
Metformin					
SHR ^1^	Male SHR	12 weeks	Metformin (500 mg/kg/day) between 4 to 12 weeks of age	Prevented hypertension	[25]
Maternal high-fructose plus post-weaning high-fat diet	Male SD ^2^ rats	12 weeks	Metformin (500 mg/kg/day) for 3 weeks during pregnancy	Attenuated hypertension;	[28]
Resveratrol and other polyphenols					
SHR	Male SHR	11 weeks	Resveratrol (50 mg/L) in drinking water between 3–11 weeks of age	Attenuated hypertension	[55]
SHR	Male and female SHR	12 weeks	Resveratrol (4g/kg of diet) between gestational day 0.5 and postnatal day 21	Attenuated hypertension	[26]
SHR	Male SHR	13 weeks	Resveratrol (50 mg/L) in drinking water between 3–13 weeks of age	Attenuated hypertension	[56]
Prenatal hypoxia and postnatal high-fat diet	Male SD rats	12 weeks	Resveratrol (4g/kg of diet) between 3–12 weeks of age	Prevented hypertension	[57]
Maternal plus post-weaning high-fructose diets	Male SD rats	12 weeks	Resveratrol (50 mg/L) in drinking water from weaning to three months of age	Prevented hypertension	[46]
Maternal plus post-weaning high-fat diets	Male SD rats	16 weeks	0.5% resveratrol in drinking water between 2 and 4 months of age	Prevented hypertension	[27]
SHR	Male SHR	7 weeks	Magnolol (100 mg/kg/day) between 4 to 7 weeks of age	Attenuated hypertension	[58]
SHR	Male SHR	20 weeks	Berberine (100 mg/kg/day) between 3 to 20 weeks of age	Attenuated hypertension and kidney damage	[59]
High-salt stroke-prone SHR	Male stroke-prone SHR	16 weeks	Genistein (0.06% wt/wt diet) between 7 to 16 weeks of age	Attenuated hypertension and kidney damage	[60]
Thiazolidinediones					
SHR	Male SHR	7 weeks	Pioglitazone (10 mg/kg/day) between 5 to 7 weeks of age	Attenuated hypertension	[61]
SHR	Male SHR	13 weeks	Rosiglitazone (150 mg/kg/day) between 5 to 13 weeks of age	Attenuated hypertension	[62]

^1^ SHR: Spontaneously hypertensive rat; ^2^ SD rats: Sprague–Dawley rats.

## Abbreviations

ACE	Angiotensin converting enzyme
AICAR	5-aminoimidazole-4carboxamide riboside
AMPK	AMP-activated protein kinase
AT1R	Angiotensin II type 1 receptor
CaMKKβ	Ca^2+^-/calmodulin-dependent protein kinase β
DOHaD	Developmental origins of health and disease
LKB1	Liver kinase B1
mTOR	Mammalian target of rapamycin
NCC	Na^+^/Cl^−^ cotransporter
NHE3	Type 3 sodium hydrogen exchanger
NKCC2	Na-K-2Cl cotransporter
PGC-1α	Peroxisome proliferator-activated receptor γ coactivator-1α
PPAR	Peroxisome proliferator-activated receptor
PPM1E	Mg^2+^-/Mn^2+^-dependent protein phosphatase 1E
PP2A	Protein phosphatase 2A
PP2C	protein phosphatase 2C
RAS	Renin-angiotensin system
SD	Sprague–Dawley
SHR	Spontaneously hypertensive rat
SIRT	Silent information regulator transcript
TAK1	TGFβ-activated kinase 1
Ulk1	Unc-51-like kinase 1
